# Klotz’s Case of Syphilitic Reinfection

**Published:** 1904-11

**Authors:** 


### Klotz's Case of Syphilitic Reinfection.

*Editor Buffalo Medical Journal:*

SIR—I wish to call your attention to what I know was an unintentional oversight, as Dr. Klotz's article appeared in the July issue of our journal.

We are striving to make our journal representative of the highest standard in American dermatology and would appreciate any help in bringing us before our special class of readers by any such occasional notices as might seem suitable to you.

A. D. MEWBORN,
*Editor Journal of Cutaneous Diseases.*

NEW YORK, October 11, 1904.

(Note by the Editor.—The foregoing refers to an error we made in crediting our reference to Klotz's case of syphilitic reinfection in the October issue of this journal. Instead of the *Journal of the American Medical Association* it should have been given to the *Journal of Cutaneous Diseases,* a mistake we greatly regret.)

